# Carcinogenesis effects of E2F transcription factor 8 (E2F8) in hepatocellular carcinoma outcomes: an integrated bioinformatic report

**DOI:** 10.1042/BSR20193212

**Published:** 2020-02-11

**Authors:** Ying Lü, Jing Zhang, Lei Li, Shun Li, Zongguo Yang

**Affiliations:** 1 Department of Integrative Medicine, Shanghai Public Health Clinical Center, Fudan University, Shanghai 201508, China; 2 Department of Surgery, Shanghai Public Health Clinical Center, Fudan University, Shanghai 201508, China; 3 Department of Laboratory Animal, Shanghai Public Health Clinical Center, Fudan University, Shanghai 201508, China

**Keywords:** disease-free survival, E2F transcription factor 8, E2F8, Hepatocellular carcinoma, overall survival, recurrence-free survival

## Abstract

This report aimed to investigate the carcinogenesis effects of E2F transcription factor 8 (E2F8) in hepatocellular carcinoma (HCC). E2F8 expression level was compared in Gene Expression Omnibus (GEO), The Cancer Genome Atlas (TCGA) and Oncomine. Survival analysis of E2F8 for HCC were conducted in Kaplan–Meier plotter. Correlations of E2F8 and clinico-pathological features were performed in TCGA. Enrichment of interacted and similar genes with E2F8 was evaluated in Gene Set Enrichment Analysis (GSEA) and Metascape. We found that E2F8 was significantly up-regulated in tumor tissues compared with nontumor tissues (all *P* < 0.01). Moreover, E2F8 was significantly overexpressed in peripheral blood mononuclear cell (PBMC) in HCC patients than that in healthy individuals (*P* < 0.001). Meta-analysis in Oncomine database confirmed that E2F8 was significantly higher in HCC tumors (*P* = 4.28E-08). Additionally, E2F8 elevation significantly correlated with overall survival (OS), recurrence-free survival (RFS), disease-specific survival (DSS) and progression-free survival (PFS) in HCC patients (all *P* < 0.01). E2F8 level was significantly higher in HCC patients with advanced neoplasm histologic grade, American Joint Committee on Cancer (AJCC) stage and α-fetoprotein (AFP) elevation (all *P* < 0.05). Cox regression model demonstrated that high E2F8 was an independent risk factor for OS and DFS in HCC patients (HR = 2.16, *P* = 0.003 and HR = 1.64, *P* = 0.002, respectively). Enrichment analysis revealed that genes interacted/similar with E2F8 were mainly enriched in cell cycle pathways/biological process. Conclusively, up-regulated in tumors, E2F8 might accelerate tumor progression and result in unfavorable outcomes in HCC patients.

## Introduction

E2F transcriptional factors implicated in the regulation of many cell possesses related to cellular proliferation, differentiation, DNA repair, cell-cycle and cell apoptosis [1,2], and were critical components of the transcriptional machinery that modulates the expression of genes required for DNA synthesis and mitosis [3]. Members of the E2F transcription factor family have been shown to be overexpressed in many types of human malignancies [4].

As one of repressors in the E2F family, E2F8 has been shown to be a suppressive regulator of transcription and cell cycle progression [2,5,6]. In addition, atypical E2F8 showed suppress effects on tumor angiogenesis in three different cancer models [7]. Conversely, emerging evidence indicated that E2F8 might also be crucially involved in promotion of carcinogenesis. Oncogenic function and therapeutic value of E2F8 have been described in lung adenocarcinoma and squamous cell carcinoma. E2F8 is overexpressed in lung cancer and enhances cell proliferation, and depletion of E2F8 inhibited cell proliferation and tumor growth [8]. E2F8 up-regulation is associated with poor prognosis in lung cancer and ovarian cancer [8–10]. Hence, the functional effects of E2F8 in human cancers remain obscure.

Recent literatures demonstrated that E2F8 is strongly up-regulated in human hepatocellular carcinoma (HCC), showed to be tumor promoter to hepatocarcinogenesis [11–13]. Aberrant overexpression of E2F8 promoted cell proliferation, enhanced colony formation and contributed to tumorigenicity in HCC cells. Mechanism analysis revealed that E2F8 influences G1–S transition of cell cycle progression, promotes the entry of S phase and mediates cyclin D1 transcription in a dominant-negative manner [14]. In contrast, E2F7 and E2F8 synchronized deletion in hepatocytes leads to HCC [15]. Considered the controversial findings and its lack of clinical investigation, we conducted an integrated bioinformatic analysis to evaluate the expression, prognostic value and potential functional mechanism of E2F8 in HCC development.

## Materials and methods

### Microarray data of GEO, TCGA and Oncomine

Microarray series of GSE45436, GSE55092, GSE60502, GSE84402, GSE33006, GSE74656 and GSE49515 were downloaded from GEO database (https://www.ncbi.nlm.nih.gov/geo/), the details of these GEO series were summarized in Table 1.

**Table 1:** Details of GEO series included in this analysis

GEO series	Contributor(s)	Tumor	Nontumor	Platform
GSE45436	Jui-Yu Hsieh, 2013	97	37	Affymetrix Human Genome U133 Plus 2.0 Array
GSE55092	Patrizia Farci, 2014	49	91	Affymetrix Human Genome U133 Plus 2.0 Array
GSE60502	KJ Kao, 2014	18	18	Affymetrix Human Genome U133A Array
GSE84402	Zhuoan Cheng, 2016	14	14	Affymetrix Human Genome U133 Plus 2.0 Array
GSE33006	Yi Huang, 2011	3	3	Affymetrix Human Genome U133 Plus 2.0 Array
GSE74656	Huiyong Yin, 2015	5	5	GeneChip® PrimeView™ Human Gene Expression Array (with External spike-in RNAs)
GSE49515	Kam Hui, 2013	10	10	Affymetrix Human Genome U133 Plus 2.0 Array

TCGA microarray data were obtained in R program using edgeR package [16]. Heatmap of E2F8 expression was performed in 50 paired tumor and nontumor samples using GraphPad prism v8.0 software (GraphPad Software, CA, U.S.A.).

Meta-analysis of E2F8 expression between HCC and normal liver tissues in Chen Liver [17] and Wurmbach Liver [18] were compared in Oncomine database. E2F8 levels with log2 median centered ratio in Chen Liver and Wurmbach Liver datasets were compared separately.

### Survival analysis

Survival analysis was performed in Kaplan–Meier plotter [19,20], which integrated both gene expression and clinical data. Patient samples were divided into two groups by median cutoff of E2F8 (RNAseq ID: 79733) to analyze the prognostic value. Kaplan–Meier survival plot with the hazard ratio (HR) with 95% confidence intervals (CI) and log rank *P* value was calculated. Outcomes including overall survival (OS), recurrence-free survival (RFS), disease-specific survival (DSS) and progression-free survival (PFS) were investigated.

### E2F8 expression comparison by clinico-pathological features

E2F8 expression data and clinical data of HCC patients in TCGA were downloaded from cBioPortal for Cancer Genomics [21,22]. We matched the gene and clinical data using VLOOKUP index in EXCEL. When those without E2F8 expression data were excluded, 361 HCC patients were included in the final analysis. All these patients were divided into E2F8 high and E2F8 low expression groups using E2F8 median cutoff.

### Enrichment analysis

Protein–protein interaction of E2F8 was conducted in Search Tool for Retrieval of Interacting Genes/Proteins (STRING) online service [23]. Interacted genes of E2F8 were also identified in Search Tool for Interacting Chemicals (STITCH) database [24]. Top 20 similar genes of E2F8 were identified in Gene Expression Profiling Interactive Analysis (GEPIA) database [25]. All these interacted genes and similar genes of E2F8 in STRING, STITCH and GEPIA were included in the enrichment analysis in Gene Set Enrichment Analysis (GSEA) molecular signatures database [26,27]. Kyoto Encyclopedia of Genes and Genomes (KEGG) pathway, Gene ontology (GO) biological process and Reactome were enriched. Metascape web service was used to validate the GO enrichment terms [28].

### Statistical analysis

Student’s *t* test or Mann–Whiney *U* test in GraphPad prism v8.0 (GraphPad Software, CA, U.S.A.) were performed to analyze the differences of gene expression. Factors associated with the survival were assessed by univariate analysis and multivariate analysis Cox regression. Only covariates significantly associated with outcomes at univariate analysis (two-sided *P* <0.10) included in the multivariate model. Results were reported as HR with 95% CI. Stata software version 16.0 (Stata Corp LLC, Texas, U.S.A.) was used. A two tailed *P* <0.05 was considered significant.

## Results

### E2F8 expression comparison

**Figure 1:**
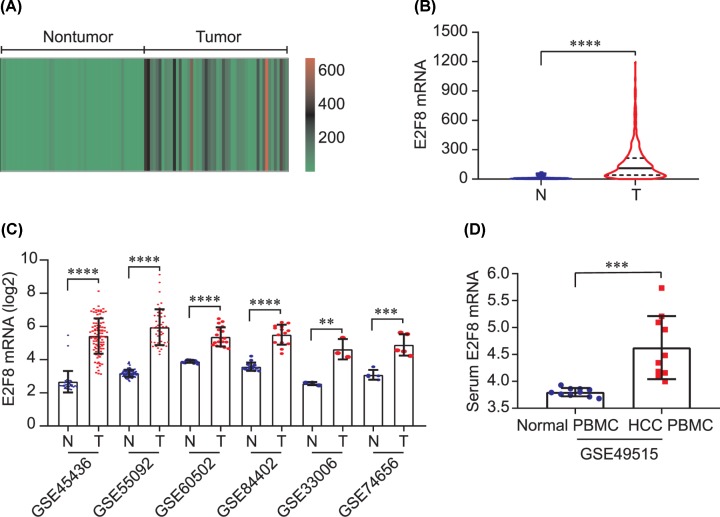
E2F8 expression in hepatocellular carcinoma (**A**) Heatmap of E2F8 mRNA expression in 50 paired tumor and nontumor tissues in TCGA dataset. (**B**) E2F8 mRNA expression in tumor and nontumor tissues in TCGA dataset. (**C**) E2F8 mRNA expression in tumor and nontumor tissues in GEO series. (**D**) E2F8 mRNA expression in serum PBMC in healthy individuals and HCC patients. **P<0.01; ***P<0.001; and ****P<0.0001.

In TCGA dataset, heatmap of E2F8 expression in 50 paired tumor and nontumor tissues was calculated, indicating that E2F8 was up-regulated in HCC tumors (Figure 1A). As shown in Figure 1B, E2F8 mRNA was significantly overexpressed in HCC tumors compared with that in nontumors (*P* < 0.0001, Figure 1B). Consistently, E2F8 mRNA was significantly up-regulated in tumor tissues than nontumors in 6 GEO series including GSE45436, GSE55092, GSE60502, GSE84402, GSE33006 and GSE74656 (all *P* < 0.01, Figure 1C). Additionally, E2F8 mRNA was also significantly elevated in serum peripheral blood mononuclear cell (PBMC) in HCC patients than that in healthy individuals (*P* < 0.001, Figure 1D).

**Figure 2:**
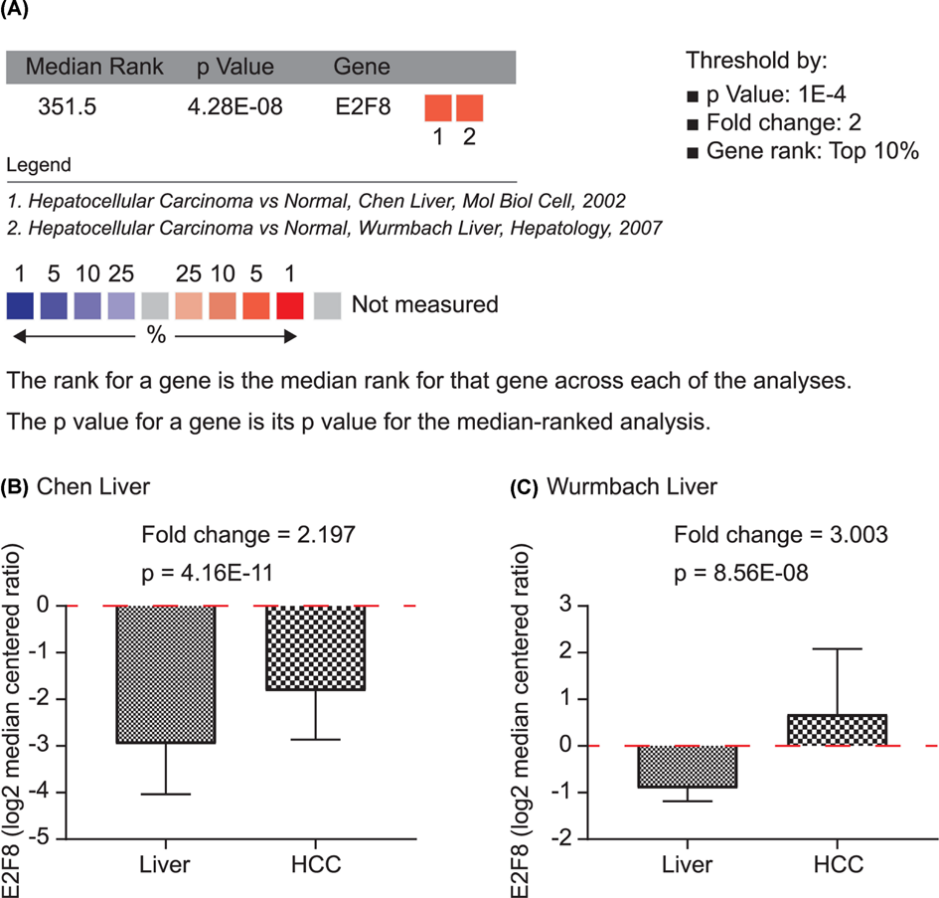
Meta-analysis of E2F8 mRNA expression in Oncomine database (**A**) Meta-analysis of E2F8 mRNA expression in two studies including Chen Liver and Wurmbach Liver in Oncomine. (**B**) E2F8 expression in normal liver and HCC in Chen Liver. (**C**) E2F8 expression in normal liver and HCC in Wurmbach Liver.

Meta-analysis of two studies in Oncomine database confirmed that E2F8 was significantly higher in HCC tumors and normal livers (*P* = 4.28E-08, Figure 2A). Separately, E2F8 mRNA was significantly overexpressed in HCC tissues than normal liver tissues in Chen Liver and Wurmbach Liver (both *P* < 0.0001, Figure 2B,C).

### OS, RFS, DSS and PFS in E2F8 high and low groups

**Figure 3:**
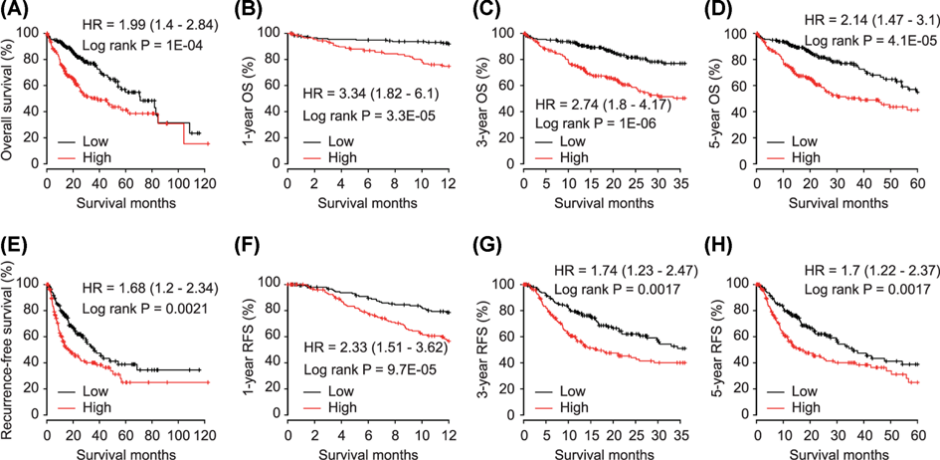
Correlation between E2F8 and overall survival (OS) and recurrence-free survival (RFS) in HCC patients (**A**) Overall survival, (**B**) 1-year OS, (**C**) 3-year OS and (**D**) 5-year OS for HCC patients grouped by E2F8 expression with median cutoff. (**E**) Recurrence-free survival, (**F**) 1-year RFS, (**G**) 3-year RFS and (**H**) 5-year RFS for HCC patients grouped by E2F8 expression with median cutoff.

As shown in Figure 3, E2F8 high expression was significantly correlated with poor OS (HR = 1.99, 95%CI = 1.4–2.84, *P* = 1E-04, Figure 3A), and same trends were observed when comparing 1-, 3- and 5-year OS in HCC patients (HR = 3.34, *P* = 3.3E-05; HR = 2.74, *P* = 1E-06 and HR = 2.14, *P* = 4.1E-05, respectively, Figure 3B–D). In addition, E2F8 overexpression was significantly associated with worse RFS in HCC patients (HR = 1.68, 95%CI = 1.2–2.34, *P* = 0.0021, Figure 3E). Also, E2F8 high level contributed to 1-year recurrence, 3-year recurrence and 5-year recurrence in HCC patients (HR = 2.33, *P* = 9.7E-05; HR = 1.74, *P* = 0.0017 and HR = 1.7, *P* = 0.0017, respectively, Figure 3F–H).

**Figure 4:**
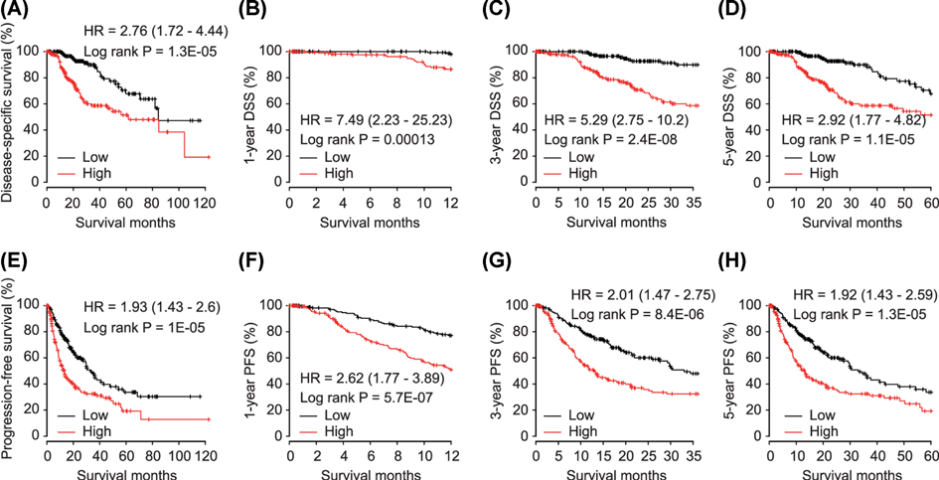
Correlation between E2F8 and disease-specific survival (DSS) and progression-free survival (PFS) in HCC patients (**A**) Disease-specific survival, (**B**) 1-year DSS, (**C**) 3-year DSS and (**D**) 5-year DSS for HCC patients grouped by E2F8 expression with median cutoff. (**E**) Progression-free survival, (**F**) 1-year PFS, (**G**) 3-year PFS and (**H**) 5-year PFS for HCC patients grouped by E2F8 expression with median cutoff.

Moreover, up-regulation of E2F8 was significantly associated with DSS, 1-, 3- and 5-year DSS in HCC patients (HR = 2.76, *P* = 1.3E-05; HR = 7.49, *P* = 0.00013; HR = 5.29, *P* = 2.4E-08 and HR = 2.92, *P* = 1.1E-05, respectively, Figure 4A–D). HCC patients with high E2F8 levels had poor PFS, 1-, 3- and 5-year PFS compared with those in E2F8 low expression group (HR = 1.93, *P* = 1E-05; HR = 2.62, *P* = 5.7E-07; HR = 2.01, *P* = 8.4E-06 and HR = 1.92, *P* = 1.3E-05, respectively, Figure 4E–H).

### Associations between E2F8 and OS and DFS in HCC patients

Based on liver hepatocellular carcinoma (TCGA, Provisional) profile in cBioPortal for Cancer Genomics, Cox regression model was used to identify the potential risk factors for OS and DFS in HCC patients. Variables including E2F8, gender, body mass index, race, tumor status, family history of cancer, pathological grade, AJCC stage, vascular invasion, AFP, new tumor event after initial treatment and hepatic inflammation were included in the univariate analysis. As summarized in Table 2, univariate analysis showed that E2F8, tumor status, AJCC stage and new tumor event after initial treatment were potential risk factors of OS in HCC patients (all *P* < 0.1, Table 2). After adjusting tumor status, AJCC stage and new tumor event after initial treatment in multivariate Cox regression model, high level of E2F8 showed to be an independent risk factor for OS in HCC patients (HR = 2.16, 95%CI = 1.3–3.59, *P* = 0.003, Table 2).

**Table 2:** Univariate and multivariate Cox regression analysis of parameters associated with OS and DFS in HCC patients^#^

Variables	OS				DFS			
	Univariate		Multivariate		Univariate		Multivariate	
	HR (95%CI)	*P* value	HR (95%CI)	*P* value	HR (95%CI)	*P* value	HR (95%CI)	*P* value
E2F8, high versus low	1.98 (1.39–2.83)	<0.001	2.16 (1.3–3.59)	0.003	1.87 (1.38–2.54)	<0.001	1.64 (1.19–2.25)	0.002
Tumor status, with tumor versus tumor free	1.59 (1.11–2.28)	0.012	2.14 (0.92– 4.95)	0.076	3.71 (2.7–5.09)	<0.001	3.65 (2.64 –5.05)	<0.001
AJCC stage								
I	Reference	1.0	Reference	1.0	Reference	1.0	Reference	1.0
II	1.49 (0.91–2.44)	0.111	1.79 (0.9–3.59)	0.098	2.03 (1.36– 3.03)	0.001	1.92 (1.26–2.91)	0.002
III	2.82 (1.85–4.28)	<0.001	4.05 (2.27–7.25)	<0.001	3.05 (2.11– 4.4)	<0.001	2.96 (2.02– 4.33)	<0.001
IV	2.64 (1.38–5.04)	0.003	2.9 (0.85–9.85)	0.088	2.07 (1.11–3.85)	0.022	1.28 (0.67–2.47)	0.454
New tumor event after initial treatment, Yes versus No	1.52 (0.95–2.42)	0.081	0.94 (0.41–2.2)	0.895	–	–	–	–

Variables including E2F8, gender, body mass index, race, tumor status, family history of cancer, pathological grade, AJCC stage, vascular invasion, AFP, new tumor event after initial treatment and hepatic inflammation were included in the univariate analysis. Only variables with *P* < 0.10 in univariate model were included in the multivariate analysis.

# Only variables significantly associated with OS/DFS in univariate analysis were presented.

Moreover, E2F8, tumor status and AJCC stage were identified as potential risk factors of DFS in HCC patients (all *P* < 0.1, Table 2). After adjusting tumor status and AJCC stage in multivariate Cox regression model, high level of E2F8 showed to be an independent risk factor for DFS in HCC patients (HR = 1.64, 95%CI = 1.19–2.25, *P* = 0.002, Table 2).

### Correlations between E2F8 and clinico-pathological features in HCC

**Figure 5:**
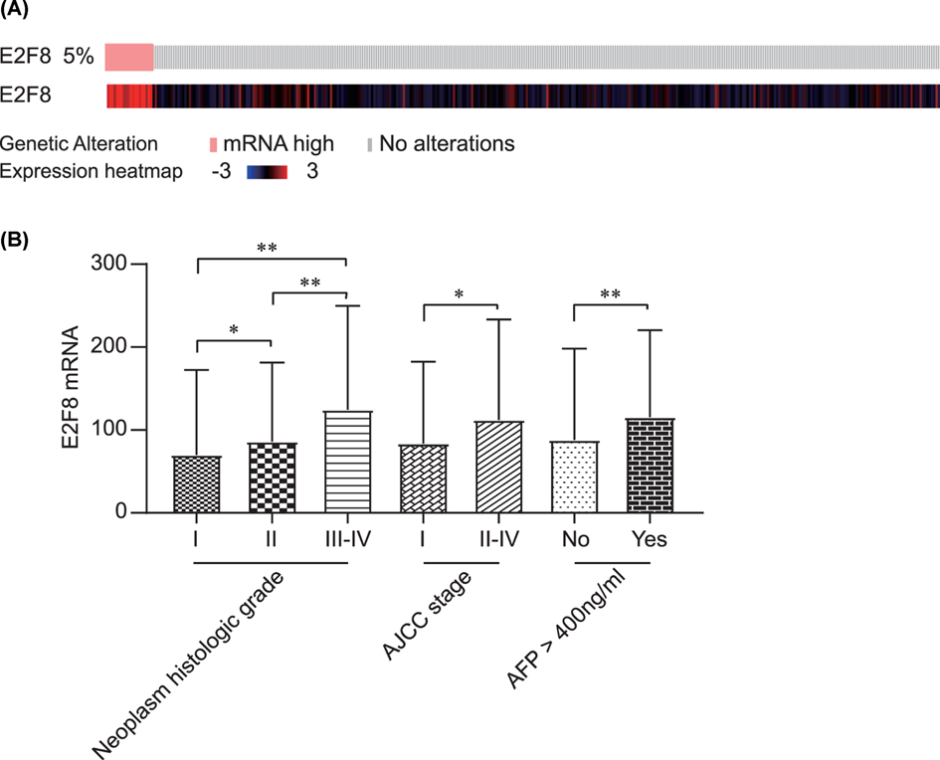
E2F8 expression heatmap and E2F8 levels by clinico-pathological features in HCC tumors (**A**) Heatmap of E2F8 mRNA in HCC samples in TCGA from cBioPortal; (**B**) E2F8 mRNA expression comparison by clinico-pathological features including neoplasm histologic grade, AJCC stage and AFP level. *P<0.05; **P<0.01.

E2F8 genetic alteration was observed in 5% of all HCC participants in TCGA Pan-Cancer Atlas (Figure 5A). Heatmap of E2F8 expression in HCC tumors was also presented in Figure 5A. As shown in Figure 5B, E2F8 mRNA was significantly higher in HCC patients with advanced neoplasm grade, AJCC stage and α-fetoprotein elevation (all *P* < 0.05, Figure 5B).

### Interacted genes and enrichment of E2F8

**Figure 6:**
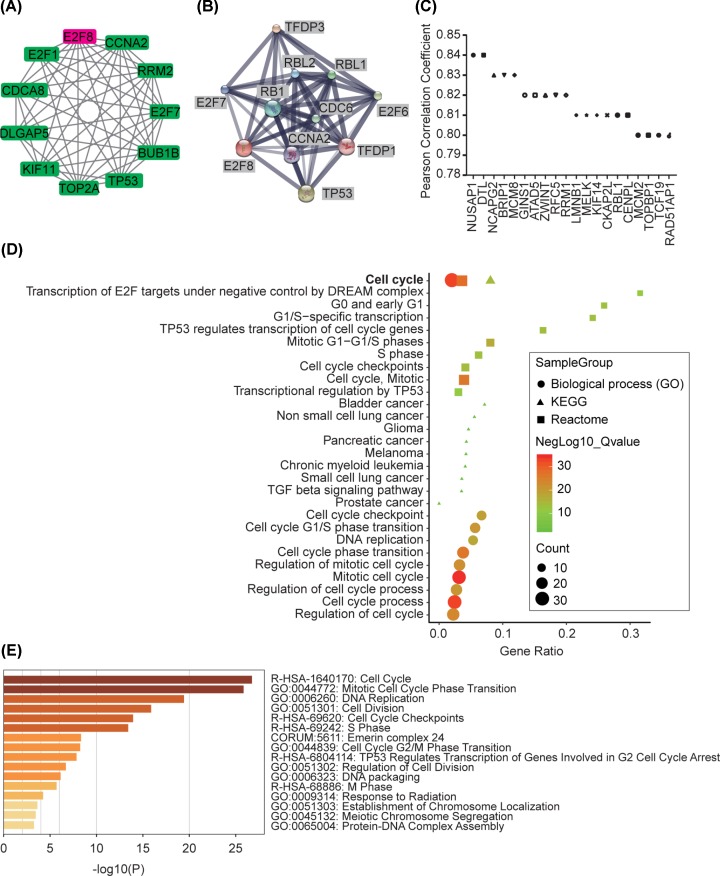
Interacted genes of E2F8 and functional enrichment (**A**) Protein–protein interaction of E2F8 in STRING. (**B**) Interacted genes with E2F8 in STITCH. (**C**) Similar genes of E2F8 in HCC tumors by GEPIA. (**D**) KEGG pathway, biological process (GO) and Reactome enrichment of interacted/similar genes with E2F8 and (**E**) Bar heatmap of enriched terms of E2F8 in Metascape.

E2F1, CDCA8, DLGAP5, KIF11, TOPA2, TP53, BUB1B, E2F7, RRM2 and CCNA2 were interacted with E2F8 in STRING database (Figure 6A), while TFDP3, TFDP1, E2F7, TP53, RBL1, RBL2, RB1, CDC6, E2F6 and CCNA2 were interacted with E2F8 in STITCH database (Figure 6B). Top 20 similar genes of E2F8 in HCC tumors in GEPIA database were identified (Figure 6C). In GSEA database, KEGG pathway, GO biological process and Reactome enrichment revealed that most genes were enriched in cell cycle pathway/biological process, mainly functioned in G and S phases (Figure 6D). In addition, these interacted genes of E2F8 also related with many types of human malignancies including bladder cancer, non-small cell lung cancer, glioma, pancreatic cancer, melanoma, chronic myeloid leukemia, small cell lung cancer and prostate cancer (Figure 6D). For enrichment validation, Metascape web service was used. Consistent with results from GSEA enrichment, enrichment heatmap demonstrated that most interacted genes or similar genes of E2F8 were involved in cell cycle regulation (Figure 6E).

## Discussion

E2F8 has been reported to overexpress in lung cancer, breast cancer, colorectal cancer, ovarian cancer, prostate cancer, esophageal squamous cell carcinoma and HCC [8,29–33]. Consistently, our results also indicated that E2F8 was up-regulated in HCC tumors and correlated with advanced tumor stage and AFP elevation. However, opposite phenomenon of cellular proliferation of E2F8 in human cancers has been reported. The ectopic E2F8 expression contributes to the suppress of E2F-targeted gene expression and slows down the proliferation of mouse embryonic fibroblasts and stress-induced skin cancer [6,34], while E2F8 overexpression promotes cell proliferation and tumorigenesis in many types of cancers including esophageal squamous cell carcinoma, papillary thyroid cancer, prostate cancer, lung cancer and liver cancer [8,14,31,32,35]. That is E2F8 might function as a transcriptional repressor or activator in cell cycle progression, even in liver development [14,15]. In HCC, considered previous evidence [36,37] and our findings, we cautiously drew hypothesis that E2F8 exerts pro-oncogenic effects in HCC progression.

Currently, the clinical significance of E2F8 in HCC aggressiveness has not yet been elucidated. Moreover, contradictory results of E2F8 in HCC tumorigenesis have been reported. Kent et al. reported that combined deletion of E2F7 and E2F8 in hepatocytes leads to HCC. Temporal-specific ablation strategies recovered that E2F8 exerts tumor suppressor effects in postnatal liver development during the first 2 weeks of life [15]. Conversely, aberrant up-regulation of E2F8 promoted cell proliferation, colony formation and tumorigenicity, while E2F8 knockdown suppressed these phenotypes in HCC cell lines [14]. E2F8 overexpression in HCC facilitated the tumor occurrence and aggressiveness through activating a E2F1/Cyclin D1 signaling pathway to regulate the G1-S transition or transcriptionally suppressing CDK1 to induce hepatocyte polyploidization. Previous evidence demonstrated that E2F8 involved closely in a variety of cellular physiological functions and pathological processes including cell cycle, cell proliferation, cell survival, DNA damage, angiogenesis, lymphangiogenesis and cell polyploidization in HCC [14,36–39]. Our enrichment analysis revealed that E2F8 and its related genes were mainly involved in regulation of cell cycle. Unfortunately, we could not conduct experimental research for investigating the oncogenic mechanisms of E2F8 in HCC. Oncogenic mechanisms of E2F8 in hepatoma cell cycle need further experimental confirmation.

In our analysis, we found that overexpression of E2F8 contributed to poor survivals in HCC, including OS, RFS, PFS and DSS. Cox hazard regression model revealed that high level of E2F8 should be an independent risk factor for OS and DFS in HCC patients. Lee et al. reported that increased expression of E2F8 is associated with prostate cancer metastasis and correlated to worse OS in prostate cancer patients [31]. In ovarian cancer, E2F8 expression levels were significantly elevated in patients with residual disease >2 cm in diameter, and E2F8 down-regulation yielded longer OS [9]. Additionally, poorer OS in non-small cell lung cancer patients with E2F8 overexpression has been observed than in those without [10]. While no significances of RFS were found both in ovarian cancer and lung cancer [9,10]. Taken together, our findings strengthened the evidence that increased E2F8 in tumors accounted for poor prognosis in HCC patients.

Intriguingly, sharp opposite results of E2F8 also have been identified in angiogenesis. Weijts et al. reported that E2F8 is essential for blood vessel formation and its deletion results in vascular defects in zebrafish and mice. Molecular mechanism demonstrated that E2F8 increased the activation of the transcription of the vascular endothelial growth factor A (VEGFA). In contrast, E2F8 deficient skin tumors displayed enhanced angiogenesis, and E2F8 inhibited tumor angiogenesis in a xenograft model for sarcomas driven by Myc and Ras oncogenes and suppressed intratumoral vessel hyperbranching via induction of delta-like ligand 4 [7].

Although the controversial effects of E2F8 existed in regulation of cell cycle progression and tumor angiogenesis, our findings supported the conclusion that E2F8 was overexpressed in tumors, correlated with advanced tumor stage and high AFP level, and poor survivals in HCC patients, indicating that E2F8 should be a potential therapeutic target for HCC treatment [36].

## Data Availability

Datasets of the current study are available from the corresponding author on reasonable request.

## Abbreviations

AFP: α-fetoprotein
AJCC: American Joint Committee on Cancer
DSS: disease-specific survival
E2F8: E2F transcription factor 8
GEO: Gene Expression Omnibus
GSEA: Gene Set Enrichment Analysis
HCC: hepatocellular carcinoma
OS: overall survival
PBMC: peripheral blood mononuclear cell
PFS: progression-free survival
RFS: recurrence-free survival
TCGA: The Cancer Genome Atlas

## References

[1] Lammens T., Li J., Leone G. and De Veylder L. (2009) Atypical E2Fs: new players in the E2F transcription factor family. Trends Cell Biol. 19, 111–118 10.1016/j.tcb.2009.01.002 19201609 PMC2808192

[2] Logan N., Graham A., Zhao X., Fisher R., Maiti B., Leone G. et al. (2005) E2F-8: an E2F family member with a similar organization of DNA-binding domains to E2F-7. Oncogene 24, 5000–5004 10.1038/sj.onc.1208703 15897886

[3] Alvarez-Fernandez M. and Malumbres M. (2015) An Atypical Oncogene Within the Atypical E2Fs. J. Natl. Cancer Inst. 107, 10.1093/jnci/djv180 PMC4836815 26089542

[4] Chen H.Z., Tsai S.Y. and Leone G. (2009) Emerging roles of E2Fs in cancer: an exit from cell cycle control. Nat. Rev. Cancer 9, 785–797 10.1038/nrc2696 19851314 PMC3616489

[5] Christensen J., Cloos P., Toftegaard U., Klinkenberg D., Bracken A.P., Trinh E. et al. (2005) Characterization of E2F8, a novel E2F-like cell-cycle regulated repressor of E2F-activated transcription. Nucleic Acids Res. 33, 5458–5470 10.1093/nar/gki855 16179649 PMC1236722

[6] Maiti B., Li J., de Bruin A., Gordon F., Timmers C., Opavsky R. et al. (2005) Cloning and characterization of mouse E2F8, a novel mammalian E2F family member capable of blocking cellular proliferation. J. Biol. Chem. 280, 18211–18220 10.1074/jbc.M501410200 15722552

[7] Weijts B., Westendorp B., Hien B.T., Martinez-Lopez L.M., Zijp M., Thurlings I. et al. (2018) Atypical E2Fs inhibit tumor angiogenesis. Oncogene 37, 271–276 10.1038/onc.2017.336 28925392 PMC5770600

[8] Park S.A., Platt J., Lee J.W., Lopez-Giraldez F., Herbst R.S. and Koo J.S. (2015) E2F8 as a Novel Therapeutic Target for Lung Cancer. J. Natl. Cancer Inst. 107, djv151 10.1093/jnci/djv151 26089541 PMC4651101

[9] Reimer D., Sadr S., Wiedemair A., Stadlmann S., Concin N., Hofstetter G. et al. (2007) Clinical relevance of E2F family members in ovarian cancer–an evaluation in a training set of 77 patients. Clin. Cancer Res. 13, 144–151 10.1158/1078-0432.CCR-06-0780 17200349

[10] Jin D.H., Kim Y., Lee B.B., Han J., Kim H.K., Shim Y.M. et al. (2017) Metformin induces cell cycle arrest at the G1 phase through E2F8 suppression in lung cancer cells. Oncotarget 8, 101509–101519 10.18632/oncotarget.21552 29254182 PMC5731892

[11] Baiz D., Dapas B., Farra R., Scaggiante B., Pozzato G., Zanconati F. et al. (2014) Bortezomib effect on E2F and cyclin family members in human hepatocellular carcinoma cell lines. World J. Gastroenterol. 20, 795–803 10.3748/wjg.v20.i3.795 24574752 PMC3921488

[12] Xanthoulis A. and Tiniakos D.G. (2013) E2F transcription factors and digestive system malignancies: how much do we know? World J. Gastroenterol. 19, 3189–3198 10.3748/wjg.v19.i21.3189 23745020 PMC3671070

[13] Zhan L., Huang C., Meng X.M., Song Y., Wu X.Q., Miu C.G. et al. (2014) Promising roles of mammalian E2Fs in hepatocellular carcinoma. Cell. Signal. 26, 1075–1081 10.1016/j.cellsig.2014.01.008 24440307

[14] Deng Q., Wang Q., Zong W.Y., Zheng D.L., Wen Y.X., Wang K.S. et al. (2010) E2F8 contributes to human hepatocellular carcinoma via regulating cell proliferation. Cancer Res. 70, 782–791 10.1158/0008-5472.CAN-09-3082 20068156

[15] Kent L.N., Rakijas J.B., Pandit S.K., Westendorp B., Chen H.Z., Huntington J.T. et al. (2016) E2f8 mediates tumor suppression in postnatal liver development. J. Clin. Invest. 126, 2955–2969 10.1172/JCI85506 27454291 PMC4966321

[16] Robinson M.D., McCarthy D.J. and Smyth G.K. (2010) edgeR: a Bioconductor package for differential expression analysis of digital gene expression data. Bioinformatics 26, 139–140 10.1093/bioinformatics/btp616 19910308 PMC2796818

[17] Chen X., Cheung S.T., So S., Fan S.T., Barry C., Higgins J. et al. (2002) Gene expression patterns in human liver cancers. Mol. Biol. Cell 13, 1929–1939 10.1091/mbc.02-02-0023 12058060 PMC117615

[18] Wurmbach E., Chen Y.B., Khitrov G., Zhang W., Roayaie S., Schwartz M. et al. (2007) Genome-wide molecular profiles of HCV-induced dysplasia and hepatocellular carcinoma. Hepatology 45, 938–947 10.1002/hep.21622 17393520

[19] Menyhart O., Nagy A. and Gyorffy B. (2018) Determining consistent prognostic biomarkers of overall survival and vascular invasion in hepatocellular carcinoma. R. Soc. Open. Sci. 5, 181006 10.1098/rsos.181006 30662724 PMC6304123

[20] Nagy A., Lanczky A., Menyhart O. and Gyorffy B. (2018) Validation of miRNA prognostic power in hepatocellular carcinoma using expression data of independent datasets. Sci. Rep. 8, 9227 10.1038/s41598-018-27521-y 29907753 PMC6003936

[21] Cerami E., Gao J., Dogrusoz U., Gross B.E., Sumer S.O., Aksoy B.A. et al. (2012) The cBio cancer genomics portal: an open platform for exploring multidimensional cancer genomics data. Cancer Discov. 2, 401–404 10.1158/2159-8290.CD-12-0095 22588877 PMC3956037

[22] Gao J., Aksoy B.A., Dogrusoz U., Dresdner G., Gross B., Sumer S.O. et al. (2013) Integrative analysis of complex cancer genomics and clinical profiles using the cBioPortal. Sci. Signal. 6, pl1 10.1126/scisignal.2004088 23550210 PMC4160307

[23] Szklarczyk D., Gable A.L., Lyon D., Junge A., Wyder S., Huerta-Cepas J. et al. (2019) STRING v11: protein-protein association networks with increased coverage, supporting functional discovery in genome-wide experimental datasets. Nucleic Acids Res. 47, D607–D613 10.1093/nar/gky1131 30476243 PMC6323986

[24] Szklarczyk D., Santos A., von Mering C., Jensen L.J., Bork P. and Kuhn M. (2016) STITCH 5: augmenting protein-chemical interaction networks with tissue and affinity data. Nucleic Acids Res. 44, D380–D384 10.1093/nar/gkv1277 26590256 PMC4702904

[25] Tang Z., Li C., Kang B., Gao G., Li C. and Zhang Z. (2017) GEPIA: a web server for cancer and normal gene expression profiling and interactive analyses. Nucleic Acids Res. 45, W98–W102 10.1093/nar/gkx247 28407145 PMC5570223

[26] Mootha V.K., Lindgren C.M., Eriksson K.F., Subramanian A., Sihag S., Lehar J. et al. (2003) PGC-1alpha-responsive genes involved in oxidative phosphorylation are coordinately downregulated in human diabetes. Nat. Genet. 34, 267–273 10.1038/ng1180 12808457

[27] Subramanian A., Tamayo P., Mootha V.K., Mukherjee S., Ebert B.L., Gillette M.A. et al. (2005) Gene set enrichment analysis: a knowledge-based approach for interpreting genome-wide expression profiles. Proc. Natl. Acad. Sci. U. S. A. 102, 15545–15550 10.1073/pnas.0506580102 16199517 PMC1239896

[28] Zhou Y., Zhou B., Pache L., Chang M., Khodabakhshi A.H., Tanaseichuk O. et al. (2019) Metascape provides a biologist-oriented resource for the analysis of systems-level datasets. Nat. Commun. 10, 1523 10.1038/s41467-019-09234-6 30944313 PMC6447622

[29] Reimer D., Sadr S., Wiedemair A., Goebel G., Concin N., Hofstetter G. et al. (2006) Expression of the E2F family of transcription factors and its clinical relevance in ovarian cancer. Ann. N. Y. Acad. Sci. 1091, 270–281 10.1196/annals.1378.073 17341621

[30] Ye L., Guo L., He Z., Wang X., Lin C., Zhang X. et al. (2016) Upregulation of E2F8 promotes cell proliferation and tumorigenicity in breast cancer by modulating G1/S phase transition. Oncotarget 7, 23757–23771 10.18632/oncotarget.8121 26992224 PMC5029661

[31] Lee S., Park Y.R., Kim S.H., Park E.J., Kang M.J., So I. et al. (2016) Geraniol suppresses prostate cancer growth through down-regulation of E2F8. Cancer Med. 5, 2899–2908 10.1002/cam4.864 27683099 PMC5083744

[32] Chang H., Song J., Wu J. and Zhang Y. (2018) E2F transcription factor 8 promotes cell proliferation via CCND1/p21 in esophageal squamous cell carcinoma. Onco. Targets Ther. 11, 8165–8173 10.2147/OTT.S180938 30532557 PMC6241692

[33] Zhang Z., Li J., Huang Y., Peng W., Qian W., Gu J. et al. (2018) Upregulated miR-1258 regulates cell cycle and inhibits cell proliferation by directly targeting E2F8 in CRC. Cell Prolif. 51, e12505 10.1111/cpr.12505 30144184 PMC6528920

[34] Thurlings I., Martinez-Lopez L.M., Westendorp B., Zijp M., Kuiper R., Tooten P. et al. (2017) Synergistic functions of E2F7 and E2F8 are critical to suppress stress-induced skin cancer. Oncogene 36, 829–839 10.1038/onc.2016.251 27452520 PMC5311251

[35] Sun J., Shi R., Zhao S., Li X., Lu S., Bu H. et al. (2017) E2F8, a direct target of miR-144, promotes papillary thyroid cancer progression via regulating cell cycle. J. Exp. Clin. Cancer Res. 36, 40 10.1186/s13046-017-0504-6 28270228 PMC5341194

[36] Lv Y., Xiao J., Liu J. and Xing F. (2017) E2F8 is a Potential Therapeutic Target for Hepatocellular Carcinoma. J. Cancer 8, 1205–1213 10.7150/jca.18255 28607595 PMC5463435

[37] Li J., Ran C., Li E., Gordon F., Comstock G., Siddiqui H. et al. (2008) Synergistic function of E2F7 and E2F8 is essential for cell survival and embryonic development. Dev. Cell 14, 62–75 10.1016/j.devcel.2007.10.017 18194653 PMC2253677

[38] Moon N.S. and Dyson N. (2008) E2F7 and E2F8 keep the E2F family in balance. Dev. Cell 14, 1–3 10.1016/j.devcel.2007.12.017 18194644

[39] Frolov M.V. and Dyson N.J. (2004) Molecular mechanisms of E2F-dependent activation and pRB-mediated repression. J. Cell Sci. 117, 2173–2181 10.1242/jcs.01227 15126619

